# The distribution of ductal carcinoma in situ (DCIS) grade in 4232 women and its impact on overdiagnosis in breast cancer screening

**DOI:** 10.1186/s13058-016-0705-5

**Published:** 2016-05-10

**Authors:** P. A. van Luijt, E. A. M. Heijnsdijk, J. Fracheboud, L. I. H. Overbeek, M. J. M. Broeders, J. Wesseling, G. J. den Heeten, H. J. de Koning

**Affiliations:** Department of Public Health, Erasmus MC, P.O. Box 2040, 3000 CA Rotterdam, The Netherlands; National Evaluation Team for Breast cancer screening in the Netherlands (NETB), Department of Public Health, Erasmus MC, University Medical Center Rotterdam, Nijmegen, The Netherlands; Department of Health Evidence, Radboud University Medical Centre, Nijmegen, The Netherlands; PALGA, the nationwide network and registry of histopathology and cytopathology in the Netherlands, Randhoeve 225 A, 3995 GA Houten, The Netherlands; National Expert and Training Centre for Breast Cancer Screening, Nijmegen, The Netherlands; Department of Radiology, Academic Medical Centre Amsterdam, Amsterdam, The Netherlands; Divisions of Diagnostic Oncology and Molecular Pathology, The Netherlands Cancer Institute, Amsterdam, The Netherlands

**Keywords:** Breast cancer, Ductal carcinoma in situ, Screening, MISCAN, Overdiagnosis

## Abstract

**Background:**

The incidence of ductal carcinoma in situ (DCIS) has rapidly increased over time. The malignant potential of DCIS is dependent on its differentiation grade.

**Methods:**

Our aim is to determine the distribution of different grades of DCIS among women screened in the mass screening programme, and women not screened in the mass screening programme, and to estimate the amount of overdiagnosis by grade of DCIS. We retrospectively included a population-based sample of 4232 women with a diagnosis of DCIS in the years 2007–2009 from the Nationwide network and registry of histopathology and cytopathology in the Netherlands. Excluded were women with concurrent invasive breast cancer, lobular carcinoma in situ and no DCIS, women recently treated for invasive breast cancer, no grade mentioned in the record, inconclusive record on invasion, and prevalent DCIS. The screening status was obtained via the screening organisations. The distribution of grades was incorporated in the well-established and validated microsimulation model MISCAN.

**Results:**

Overall, 17.7 % of DCIS were low grade, 31.4 % intermediate grade, and 50.9 % high grade. This distribution did not differ by screening status, but did vary by age. Older women were more likely to have low-grade DCIS than younger women. Overdiagnosis as a proportion of all cancers in women of the screening age was 61 % for low-grade, 57 % for intermediate-grade, 45 % for high-grade DCIS. For women age 50–60 years with a high-grade DCIS this overdiagnosis rate was 21–29 %, compared to 50–66 % in women age 60–75 years with high-grade DCIS.

**Conclusions:**

Amongst the rapidly increasing numbers of DCIS diagnosed each year is a significant number of overdiagnosed cases. Tailoring treatment to the probability of progression is the next step to preventing overtreatment. The basis of this tailoring could be DCIS grade and age.

## Background

Ductal carcinoma in situ (DCIS) is a “neoplastic proliferation of cells within the ductal-lobular structures of the breast that has not penetrated the myoepithelial-basement membrane interface” [1]. Before the introduction of mammography screening, DCIS was rarely diagnosed. In 1989, 366 women in the Netherlands were diagnosed with DCIS. In 2003, more than 10 years after the introduction of mass screening, 1171 women had a DCIS diagnosed. With the introduction of digital screening this figure rose to 2046 women in 2011, and most recently to 2406 in 2014 [2].

The extent to which DCIS represents overdiagnosis has been extensively debated in relation to organised screening programmes [3–6]. Overdiagnosis is defined as a lesion diagnosed by screening in an asymptomatic woman that would not have been detected during the woman’s lifetime in the absence of screening [4]. To predict the probability of a DCIS to progress to invasive carcinoma, six different grading systems were proposed, based on morphology or molecular profile [7]. All of these classify DCIS into three categories of malignant potential: low (I), intermediate (II), or high (III). The grade of DCIS is correlated with the risk of progression, as well as with the grade of concurrent invasive carcinoma [8–13]. The transition from low-grade DCIS to high-grade DCIS or to high-grade invasive carcinoma is deemed unlikely [8–10, 12].

The grade distribution of DCIS has been studied in mostly small series [6, 14–18], or only included screen-detected cases (Table 1) [19]. More insight in this distribution based on larger numbers in both screened and non-screened populations is of paramount importance and may improve our estimates of overdiagnosis.

**Table 1:** Grade distribution by detection mode and sample size compared to previous studies on DCIS grade distribution

	Screen detected				Symptomatic				Not specified			
	DCIS grade 1	DCIS grade 2	DCIS grade 3		DCIS grade 1	DCIS grade 2	DCIS grade 3		DCIS grade 1	DCIS grade 2	DCIS grade 3	
	%	%	%	N	%	%	%	N	%	%	%	N
This study	16	32	52	1430	19	28	54	263				
Evans 2001 [6]	13	18	69	222	16	23	61	151	-	-	-	-
Kessar 2002 [15]	23	23	54	98	19	19	62	52	-	-	-	-
Meijnen 2005 [16]	22	31	47	87	26	24	49	293	-	-	-	-
de Roos 2007 [14]	7	44	53	54	30	45	25	20	-	-	-	-
Sorum 2010 [18]	-	-	-	-	-	-	-	-	23	23	53	2403
Bluekens 2012 [19]	15	32	54	853	-	-	-	-	-	-	-	-
Weigel 2015 [33]	18	39	42	898	-	-	-	-	-	-	-	-

*DCIS* ductal carcinoma in situ

The aim of this study was to establish the distribution of different grades of DCIS in different subgroups based on mass screening status and age group, and to estimate the overdiagnosis rate for each grade and age group specifically.

## Methods

### Patient selection

We obtained 17,744 excerpts from 12,301 women with DCIS from the years 2007, 2008 and 2009 from the ‘Nationwide network and registry of histopathology and cytopathology in the Netherlands’ (PALGA). PALGA is a national database containing the excerpts and coded diagnoses of all pathological and cytological examinations performed in the Netherlands [20]. The mass screening status of these women was established by linking the database to the databases of the screening organisations by an independent third party, with the permission of the screening organisations. Our database contained anonymised records of mass screening status (positive, negative, year of last mass screening and number of mass screening examinations), age, year of diagnosis, and a short summary of the conclusion of the original pathology report.

From the 12,301 women, we excluded those who also had a concurrent invasive breast cancer (ipsilateral or contralateral, N = 7089), those who had a lobular carcinoma in situ and no DCIS (N = 6), those who turned out after excision biopsy or ablation not to have any malignancy (N = 131), those who had recently been treated for invasive breast cancer (N = 247), those who had no grade mentioned in the excerpt (N = 17), those who had an inconclusive excerpt on invasion or otherwise (N = 242), and women who had a prevalent DCIS, rather than a new diagnosis in the study period (N = 354). We excluded contralateral disease because our model does not include bilateral disease.

### DCIS detected by mass screening

DCIS were assumed to be ‘detected by mass screening’ when a woman had had a positive screening examination between 2007 and 2009. Women who had participated in the screening programme, and did not have any positive screens, but who did have a DCIS diagnosis in 2007, 2008, or 2009 were assumed to have an interval DCIS. The number of interval DCIS increased across the study period due to the cumulative effect of interval DCIS diagnosed in women screened in the previous year (2007) or in the 2 previous years (2007 and 2008). Interval DCIS were rare in 2007 because of the low frequency of interval carcinomas within the same calendar year in which the screening examination took place. In 2008, interval DCIS were diagnosed in women screened in 2007 or 2008, and in 2009, interval DCIS were diagnosed in women screened in 2007, 2008 or 2009.

Women who were not known to the screening organisations may have been under clinical surveillance because of high familial risk, frequent (benign) breast anomalies, or because of personal preference. Diagnoses in this group may be the result of screening, but are not the result of the mass screening programme. Therefore we cannot conclude that DCIS not detected by mass screening, were not detected by screening. To compare the distribution of DCIS detected by mass screening to DCIS not detected by mass screening, we, therefore, chose to compare the DCIS detected by mass screening to the interval DCIS.

### Grading of DCIS

In line with the Dutch guidelines, the classification by Holland et al. is almost exclusively used [21]. At the start of the mass screening programme in the early 1990s, pathologists were instructed on how to uniformly classify each DCIS.

DCIS grade was determined using the information in the short summary of the pathology report by description, i.e. high, moderate, or low differentiation; low, intermediate, or high malignancy potential; or grade I, II, or III. If the summary contained more than one grade, this case was graded according to the highest grade mentioned. If there was a discrepancy between grades in different specimens of the same patient, the grade was based on the most representative specimen, i.e. resection is more representative than biopsy, but biopsy is more representative than cytology.

### Statistical analysis

Proportions of DCIS grades were calculated by year, age group, and screening status. We compared these proportions between screening groups using the Pearson chi-square test. Multivariate analyses on age groups were performed with a logistic regression model. The statistically significant parameters were identified by the introduction of variables in a stepwise manner. All calculations were performed using IBM SPSS version 20.0 (IBM Corp., Armonk, NY, USA).

### Modelling approach

**Fig. 1:**
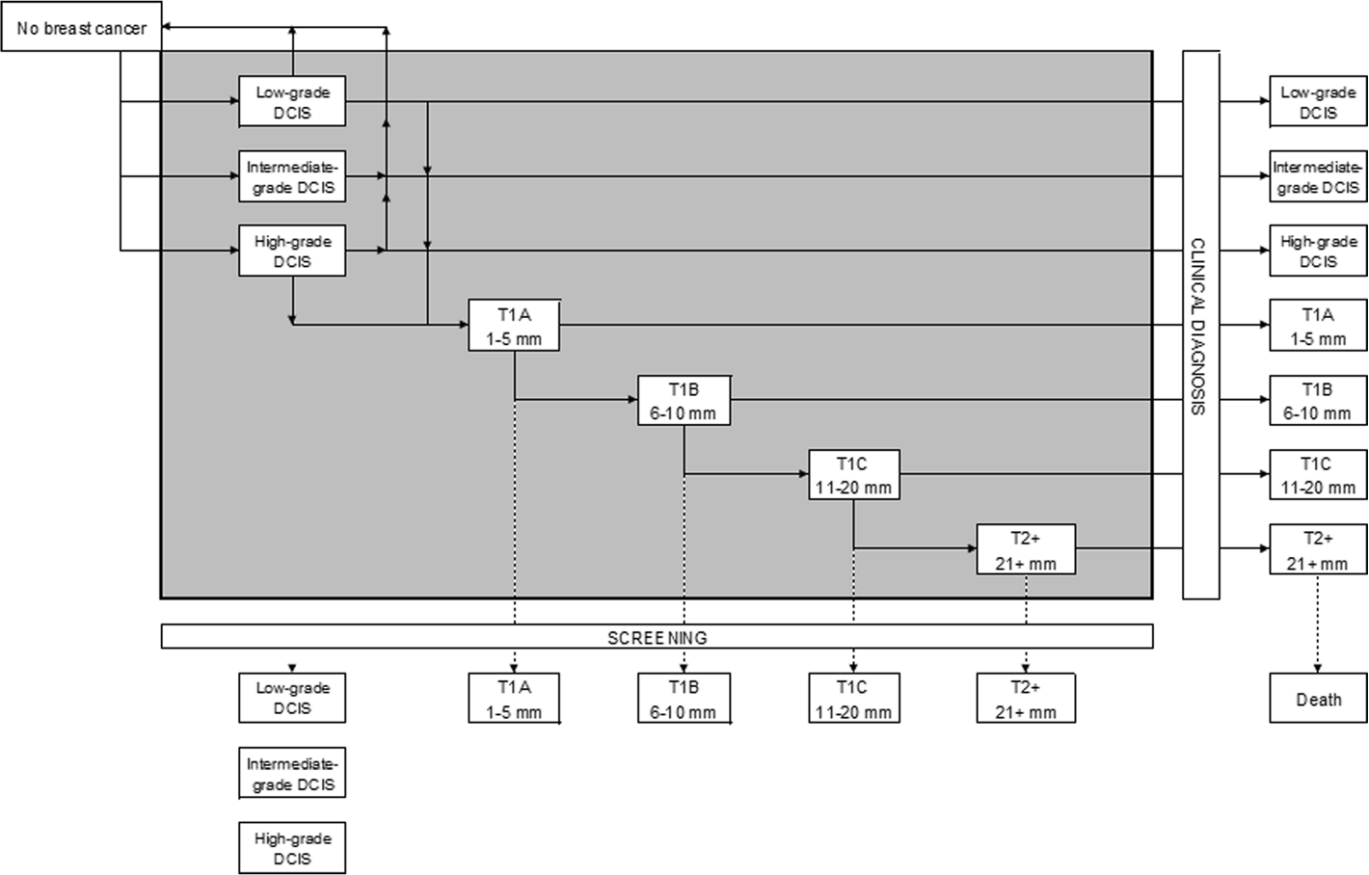
Schematic drawing of the extended MISCAN model. Transition possibilities are indicated with *arrows*. All diseases within the *grey area* are preclinical disease, after diagnosis they are either clinically detected or detected by mass screening. There is no transition between low-grade DCIS, intermediate-grade DCIS and high-grade DCIS. *DCIS* ductal carcinoma in situ, *MISCAN* MIcrosimulation SCreening ANalysis (predicted rates by the model), *T1a* tumour with a diameter up to 5 mm, *T1b* tumour with a diameter from 5 mm up to 10 mm, *T1c* tumour with a diameter from 10 mm up to 20 mm, *T2 +* any tumour with a diameter larger than 20 mm

The MISCAN model is a microsimulation model that simulates the individual life histories of women [22]. The probability of each woman to have an onset of breast cancer is determined by calibrating the model to the incidence rate in 1989 (the year before screening was introduced), adjusted with an annual percentage change of 1.4 % to account for the rising background breast cancer incidence [23]. The natural history of breast cancer is modelled as a Markov-like progression through the successive preclinical stages of the disease. Details of the model have been described previously [4]. For this analysis we added the three DCIS grades to the model, using the age-dependent grade distribution found in this study (Fig. 1).

Following onset, breast cancer in a preclinical stage can progress to the next preclinical stage (dependent on the duration of the previous state), or become clinically detected. In addition, the DCIS stages may also regress to normal [24, 25]. Screening is superimposed on this life history.

The transition probabilities, duration of tumour stages, and test sensitivities were calibrated using data from the Dutch population and Dutch breast cancer screening from 1975 to 2010 on breast cancer incidence by stage, age, and detection mode. The Dutch nationwide breast-cancer screening programme has invited all women aged 50–69 since 1990 and women aged 50–75 since 1998 biennially for a mammographic screening examination, free of charge. The attendance rate is approximately 80 % [26].

**Fig. 2:**
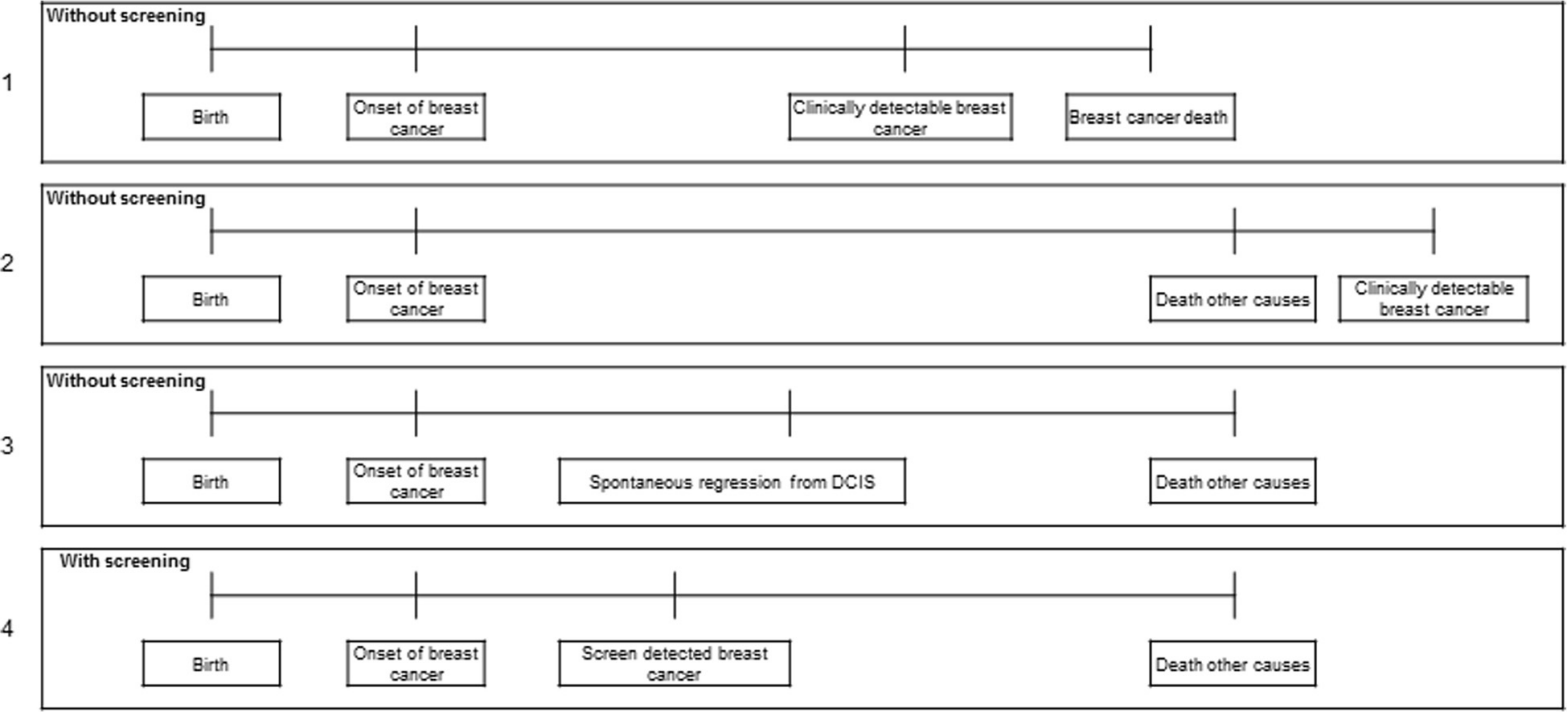
Screening affecting three women differently. The *first box* is the life history of a woman who has an onset of breast cancer, is diagnosed clinically, and dies of breast cancer. The *second box* is the life history of a woman who also has an onset of breast cancer, but who dies of other causes before this would be detected. The *third box* is the life history of a woman who has an onset of breast cancer, but also a spontaneous regression, this woman would not have been diagnosed without screening. The *fourth box* indicates the situation for these three women had screening been introduced. The woman in the *first box* no longer dies from breast cancer; the other two women do not benefit from screening, they have been overdiagnosed

We chose to look at model outcomes for the years 2000–2009 because there was a steady state situation in these years, more than 10 years after the start of the screening programme. We evaluated the following output: incidence rate by detection mode (screen detected or clinically detected), age, and year of diagnosis. The model compares women in the situation with screening, to the same women in the situation without screening; if a woman has a screen-detected cancer, but would not have had a diagnosis in the situation without screening, this case is regarded as overdiagnosed (Fig. 2).

The estimates and definitions of overdiagnosis vary widely among international publications [4]. To minimise confusion, we used the definitions of overdiagnosis which were deemed most useful by an independent review panel in the UK; from a population perspective: the proportion of all cancers ever diagnosed in women of the screening age and over (50–100 years) that are overdiagnosed; and from an individual perspective: the proportion of all cancers ever diagnosed in women of the screening age (50–75 years) that are overdiagnosed [27].

### Assumptions on natural behaviour of DCIS

In the original model a 2 % regression rate, an 11 % progression rate, and a 5 % clinical detection rate was assumed for all DCIS, resulting in a proper fit of incidence [28]. Little is known about the natural history of DCIS without treatment. Small studies were published, indicating a progression rate of one in two to one in three for low-grade DCIS, one in three for intermediate-grade DCIS and two in three in high-grade DCIS [29, 30]. Progression rate may differ from the rate assumed in the original model. In the new model we assumed that intermediate-grade DCIS has the same transition probabilities as all DCIS had in the original model. We lowered the regression rate to 1 % for high-grade DCIS, and increased the regression rate to 4 % for low-grade DCIS, based on the findings of Sanders et al. [30]. The probability for a DCIS to be clinically detected was assumed independent of grade. The probability of progression: 16 % for low-grade DCIS, 31 % for intermediate-grade DCIS, and 53 % for high-grade DCIS, was estimated by correcting the probabilities of low-grade DCIS and high-grade DCIS by the progression found in literature [29, 30]. Adjusting the progression rate and therefore the duration of the state, influences all successive states, because the progression of each successive state is dependent on the duration of the previous state. High-grade invasive breast cancer follows high-grade DCIS and low-grade invasive breast cancer follows low-grade DCIS. We calibrated DCIS incidence rate to observed data for the period 1990–2010.

## Results

### Patients/distribution of DCIS grade

Patient characteristics are summarised in Table 2. There was no significant difference in the distribution of grades between the DCIS detected by mass screening and the DCIS not detected by mass screening (from the interval group); 16.4–18.8 % were low grade, 27.2–31.6 % were intermediate grade, and 52.0–54.0 % were high grade (Table 3).

**Table 2:** Descriptive statistics of the DCIS cases reviewed

	Known at mass screening		Not known at mass screening		*P* value
	N	%	N	%	
Patients	4.075		8.226		
Exclusions	2.382	58 %	5.687	69 %	
Inclusions	1.693	42 %	2.539	31 %	
Year diagnosis					
2007	429	25 %	865	34 %	<0.001
2008	583	34 %	806	32 %	
2009	681	40 %	868	34 %	
Age group					
<49	0	0 %	651	26 %	<0.001
49–75	1.690	100 %	1.686	66 %	
>75	3	0 %	202	8 %	
Screen result					
Positive screen	1.430		n.a.		
No positive screen	263		n.a.		
	Mean		Mean		
Age	60.8		56.3		<0.001

‘Known at mass screening’ are all women who were listed in the database of the screening organisations with a positive or a negative screen, ‘Not known at mass screening’ are all women who were not mentioned in the screening organisation’s database

*DCIS* ductal carcinoma in situ, *n.a*. not applicable

**Table 3:** Distribution of different DCIS grades by screening status and age group

		Detected at mass screening		Screen negative		*P* value
		N	%	N	%	
Age group						
<49		0		0		
	Low-grade DCIS	0	n.a.	0	n.a.	
	Intermediate-grade DCIS	0	n.a.	0	n.a.	
	High-grade DCIS	0	n.a.	0	n.a.	
49–75		1429		261		
	Low-grade DCIS	234	16.4 %	49	18.8 %	
	Intermediate-grade DCIS	452	31.6 %	71	27.2 %	0.579
	High-grade DCIS	743	52.0 %	141	54.0 %	
>75		1		2		
	Low-grade DCIS	0	0.0 %	0	0.0 %	
	Intermediate-grade DCIS	0	0.0 %	2	100.0 %	0.297
	High-grade DCIS	1	100.0 %	0	0.0 %	

The *P* values indicate the significance of the difference of these distributions between screening status. Low-grade DCIS: DCIS with a low malignant potential. Intermediate-grade DCIS: DCIS with an intermediate malignant potential. High-grade DCIS: DCIS with a high malignant potential

*DCIS* ductal carcinoma in situ, *n.a.* not applicable

Univariate analysis of the group, not detected by mass screening, showed that DCIS grade has an inverse linear association with 5-year age group (*P* value = 0.015), and with age as a linear variable (*P* value = 0.018). Year of diagnosis did not contribute in this group. Overall the year of diagnosis was a significant independent variable (*P* value = 0.02) (Table 4).

**Table 4:** Distribution of different DCIS grades by year and screening status

		Detected at mass screening		Screen negative		*P* value
		N	%	N	%	
Year						
2007		410		19		
	Low-grade DCIS	59	14.4 %	3	15.8 %	
	Intermediate-grade DCIS	109	26.6 %	2	10.5 %	0.083
	High-grade DCIS	242	59.0 %	14	73.7 %	
2008		525		58		
	Low-grade DCIS	91	17.3 %	11	19.0 %	
	Intermediate-grade DCIS	167	31.8 %	15	25.9 %	0.827
	High-grade DCIS	267	50.9 %	32	55.2 %	
2009		495		186		
	Low-grade DCIS	84	17.0 %	35	18.8 %	
	Intermediate-grade DCIS	176	35.6 %	56	30.1 %	0.651
	High-grade DCIS	235	47.5 %	95	51.1 %	

The *P* values indicate the significance of the difference of these distributions between screening status. Low-grade DCIS: DCIS with a low malignant potential. Intermediate-grade DCIS: DCIS with an intermediate malignant potential. High-grade DCIS: DCIS with a high malignant potential

*DCIS* ductal carcinoma in situ

### Estimating overdiagnosis

**Fig. 3:**
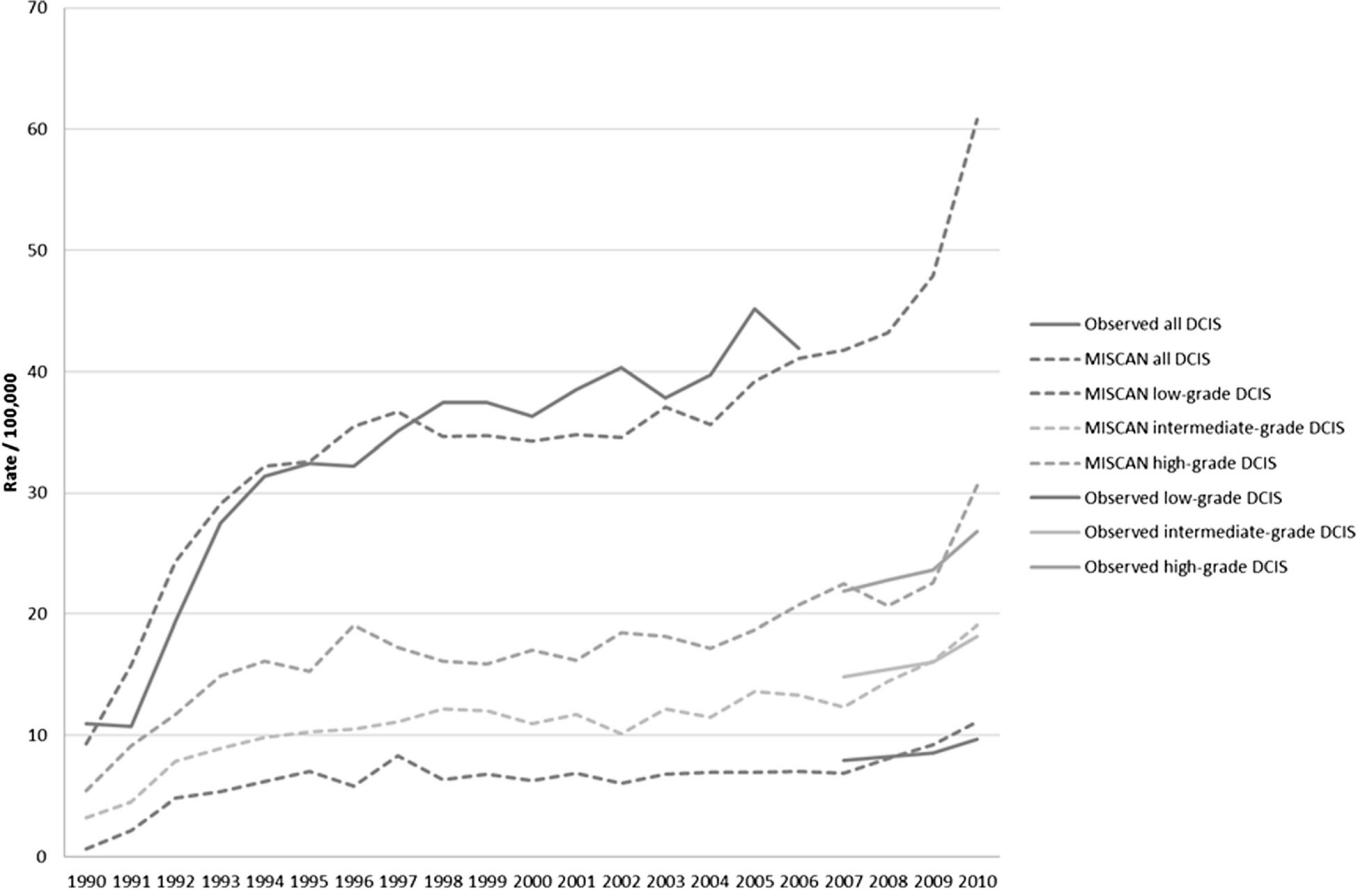
Low-grade DCIS, intermediate-grade DCIS and high-grade DCIS per 100,000 women aged 50–60. Observed: the number of DCIS as calculated when applying DCIS grade distribution to the data on total DCIS incidence from the Dutch Cancer Registry. DCIS ductal carcinoma in situ, MISCAN MIcrosimulation SCreening ANalysis (predicted rates by the model)

The distribution of DCIS grade was included in the model and the new model was calibrated estimating dwell times and probabilities of transition on incidence data from the Cancer Registry and grade distribution from our study (Fig. 3).

Overdiagnosis estimates from the model were, from the population perspective: 60 % of low-grade DCIS, 56 % of intermediate-grade DCIS, 45 % of high-grade DCIS. Overdiagnosis estimates from the individual perspective were: 61 % of low-grade DCIS, 57 % of intermediate-grade DCIS, 45 % of high-grade DCIS. When stratified by age group, the younger women had a much lower overdiagnosis rate when being diagnosed with a high-grade DCIS, varying from 21 % in age group 50–55 to 29 % in age group 55–60, up to 66 % in age group 70–75 (Table 5).

**Table 5:** Overdiagnosis estimates by two different definitions

	Low-grade DCIS	Intermediate-grade DCIS	High-grade DCIS
Population perspective	60 %	56 %	45 %
Individual perspective	61 %	57 %	45 %
Individual perspective by age group			
50–55	58 %	46 %	21 %
55–60	62 %	55 %	29 %
60–65	66 %	64 %	50 %
65–70	49 %	52 %	61 %
70–75	54 %	58 %	66 %

Population perspective: the proportion of all cancers ever diagnosed in women of the screening age and over (50–100 years) that are overdiagnosed. Individual perspective: the proportion of all cancers ever diagnosed in women of the screening age (50–75 years) that are overdiagnosed. Low-grade DCIS: DCIS with a low malignant potential. Intermediate-grade DCIS: DCIS with an intermediate malignant potential. High-grade DCIS: DCIS with a high malignant potential

*DCIS* ductal carcinoma in situ

## Discussion

This is the largest study on the distribution of DCIS grade and the first modelling study to estimate overdiagnosis rate by DCIS grade. The distribution of grades in DCIS is dependent on age, but not on mass screening status. This is in accordance with earlier studies on grade distribution. The overall distribution is also consistent with these studies (Table 4) [6, 14–16, 18, 19, 31].

The incidence rate of DCIS has increased rapidly over recent years. DCIS is unequivocally associated with mammography screening. Approximately one third of the cases in the database were detected by mass screening, which corresponds to the overall distribution of breast cancers detected by mass screening (both in situ and invasive) of all breast cancers in the Dutch population, and to the findings of Shin et al. [32]. However, in our study, when linking Dutch pathology reports to the records of the screening organisations, most DCIS were not known at mass screening organisations. This can partly be explained by the fact that one of the nine organisations that were responsible for screening at the time did not deliver data to be linked to the PALGA database. This organisation represents approximately 15 % of all screened women annually. Second, we do not know how the diagnoses not detected by mass screening were established. Given the age distribution and the fact that DCIS is generally not palpable, we assume that the majority of these cases are diagnosed through screening outside the mass screening programme.

As expected, and in line with previous studies, we found more low-grade DCIS in older women [33]. In general, more aggressive cancers are diagnosed earlier in life. Those that remain for detection at an older age are more likely to be less aggressive [34].

In the Netherlands, a transition to screening with digital mammography was made between 2005 and 2010. In 2010, the detection rate of DCIS in mass screening increased substantially, probably as a result of the introduction of digital mammography screening. Currently, it is not yet clear whether this is a prevalence effect or a lasting effect. We studied the years 2007, 2008 and 2009; thus, an increasing proportion of the DCIS we considered has been found with digital screening. We have no knowledge which DCIS were detected by digital mammography or film screen mammography. Also, the DCIS detected outside the mass screening programme are equally likely to have been detected with digital mammography. We did not find a difference in grade distribution in screen-detected DCIS over this period; therefore it seems unlikely that digital screening will have significantly altered the grade distribution, which is also in accordance with the findings of Bluekens et al. [19].

We have found that grade distribution for DCIS in the years 2007, 2008 and 2009, was inversely related to age, but we have no information on historical development of this distribution. For our study, we assumed the distribution to be stable over time.

Considerable controversy exists on whether DCIS is the ideal stage of the disease for early detection, or whether the detection of DCIS represents overdiagnosis, and, consequently, overtreatment. However, agreement exists that it is essential to determine which individual diagnosis is overdiagnosis and which is not. Central to this discussion is the natural behaviour of DCIS. Now that we have specified grades of DCIS in the microsimulation model, we can estimate overdiagnosis more accurately. Only 16.4 % of DCIS detected by mass screening are low grade, 60 % respectively 61 % of which are overdiagnosed, depending on the definition of overdiagnosis. We found that 50.9 % of all DCIS detected by mass screening are high grade, and therefore have a high risk of progression. In these cases we are bound to find aggressive cancer earlier and to prevent fast-growing invasive cancer, but even so, 45 % of these cases are overdiagnosed, independent on the definition of overdiagnosis. For younger women (age 50–60) with a high-grade DCIS however, overdiagnosis estimates vary between 21 % and 29 % from an individual perspective, therefore for these women screening is most protective.

We found an increasing amount of overdiagnosis in older women with high-grade DCIS; this is the result of a longer dwell time in the model in high-grade DCIS in women over 60. This dwell time was calibrated by the model. A disease with a longer dwell time is more likely to be detected by screening. The longer dwell time of high-grade DCIS in older women correlates to the findings of Weigel et al., who found a higher detection rate of high-grade DCIS in older women [33].

Our overdiagnosis estimates make a general decision on treatment from a population-based approach a very difficult one for women with DCIS. We estimate that 60 % of these women would be overtreated if they undergo treatment for this disease, of which they would never have been aware in the absence of screening. On the other hand, they are diagnosed with an entity that carries a specific risk for progression to an invasive and potentially lethal disease and will therefore lean towards treatment, rather than active surveillance. If this entity would be named differently this might be perceived differently [35]. DCIS can also be regarded as a risk factor like lobular carcinoma in situ (LCIS). One can question whether the increased risk in DCIS, as compared to LCIS, justifies the current practice of invasive treatments.

Specific estimates for overdiagnosis rate by grade will become increasingly important. These estimates may change when the treatment for DCIS can be even more customised according to grade [36]. To our knowledge, a trial to compare treatment of DCIS to active surveillance is planned [37].

### Limitations of the study

We did not review grading or examine inter-observer variation between pathologists, because this was beyond the scope of our study. PALGA and the Dutch association of pathologists will be conducting a study to evaluate the inter-observer variation in the near future. We believe our study to be a proper representation of the current Dutch situation. There is no reason to suspect that DCIS not detected by mass screening represents a different patient group than DCIS detected by mass screening, and for that reason, for both groups the same dilemma with regard to a possible inter-observer variation exists.

Assumptions on behaviour of DCIS were done on older studies. Advances have been made in the evaluation of biopsies. Currently more sampling is done and pathologists are more aware of the possible findings in DCIS, this could influence the assumptions on behaviour of DCIS if the studies on which they are based were repeated now.

## Conclusions

DCIS grade is almost equally distributed across the screened population in the breast cancer screening programme and the population not subjected to/participating in mass screening.

DCIS has been divided into three grades, each constituting a unique entity with its own natural history. We found that the distribution of these grades is not dependent on mass screening status, but is dependent on age. When taking the different grades into account, overdiagnosis rates of breast cancer in mass screening are 60 % for low-grade DCIS and 45 % for high-grade DCIS from a population perspective, and 61 % and 45 % respectively from an individual perspective. When taking the younger ages and high grade into account overdiagnosis rate from an individual perspective is 21–29 %.

These figures underline the necessity of large randomised trials for watchful waiting in low-grade DCIS, whether these are detected in a mass screening programme or not.

### Ethics statement

Since the research was retrospectively performed on data, and did not involve subjecting patients to certain acts or appointing them behavioural changes, consent from the medical ethics commission was not required according to Dutch law. We only ever received fully anonymised data.

### Consent statement

By participating in the programme, women automatically consent to the use of their data to evaluate and improve the programme. Information about the use of data is provided with a flyer accompanying the invitation letter. If a woman does not want the screening organisation to use her data for this purpose, she can return the signed corresponding form to the screening organisation. Only a minor fraction (0.01 %) used this possibility.

## Abbreviations

DCIS: ductal carcinoma in situ
LCIS: lobular carcinoma in situ
MISCAN: MIcrosimulation SCreening ANalysis
PALGA: the nationwide network and registry of histopathology and cytopathology in the Netherlands
